# Sea Sickness

**Published:** 1879-10

**Authors:** 


SEA SICKNESS.—The opinion so commonly held in regard to sea sickness, namely, that it is due either to a congestion of the brain or to a commotion in the abdominal viscera, caused by the motion of the vessel, are very plausibly combatted by M. Pellerin, who, in a paper read before the French Academy, attributes the malady to a deranged circulation of the blood, produced by the alternate rolling and heaving of the vessel. The result of this, he says, is not a congestion of the brain, which is, on the contrary, deprived of some of the blood required to keep up a stimulus of that nervous centre—that sensation which is felt in sea sickness resembling peculiarly what is felt immediately after a letting of the blood when the patient sits or stands, namely, a disposition to vomit, or actual vomiting. In support of this opinion, mention is made of the fact that persons who are liable to sea sickness experience its effects in a much slighter degree when they are in a horizontal position—the relief thus afforded being like that which is produced in the same position when a person is in a state of syncope.

